# Global Threat of Carbapenem-Resistant Gram-Negative Bacteria

**DOI:** 10.3389/fcimb.2022.823684

**Published:** 2022-03-15

**Authors:** Shio-Shin Jean, Dorji Harnod, Po-Ren Hsueh

**Affiliations:** ^1^ Department of Emergency and Critical Care Medicine, Min-Sheng General Hospital, Taoyuan, Taiwan; ^2^ Department of Pharmacy, College of Pharmacy and Health care, Tajen University, Pingtung, Taiwan; ^3^ Division of Critical Care Medicine, Department of Emergency and Critical Care Medicine, Shuang Ho Hospital, Taipei Medical University, New Taipei City, Taiwan; ^4^ Department of Emergency, School of Medicine, College of Medicine, Taipei Medical University, Taipei, Taiwan; ^5^ Departments of Laboratory Medicine and Internal Medicine, China Medical University Hospital, School of Medicine, China Medical University, Taichung, Taiwan; ^6^ School of Medicine, China Medical University, Taichung, Taiwan; ^7^ Ph.D Program for Aging, School of Medicine, China Medical University, Taichung, Taiwan; ^8^ Departments of Laboratory Medicine and Internal Medicine, National Taiwan University Hospital, National Taiwan University College of Medicine, Taipei, Taiwan

**Keywords:** carbapenem-resistant, extensively-drug resistant, gram-negative bacteria, ceftazidime-avibactam, enterobacterales, *Pseudomonas aeruginosa*, *Acinetobacter baumannii* complex

## Abstract

Infections caused by multidrug-resistant (MDR) and extensively drug-resistant (XDR) Gram-negative bacteria (GNB), including carbapenem-resistant (CR) Enterobacterales (CRE; harboring mainly *bla*_KPC_, *bla*_NDM_, and *bla*_OXA-48_-like genes), CR- or MDR/XDR-*Pseudomonas aeruginosa* (production of VIM, IMP, or NDM carbapenemases combined with porin alteration), and *Acinetobacter baumannii* complex (producing mainly OXA-23, OXA-58-like carbapenemases), have gradually worsened and become a major challenge to public health because of limited antibiotic choice and high case-fatality rates. Diverse MDR/XDR-GNB isolates have been predominantly cultured from inpatients and hospital equipment/settings, but CRE has also been identified in community settings and long-term care facilities. Several CRE outbreaks cost hospitals and healthcare institutions huge economic burdens for disinfection and containment of their disseminations. Parenteral polymyxin B/E has been observed to have a poor pharmacokinetic profile for the treatment of CR- and XDR-GNB. It has been determined that tigecycline is suitable for the treatment of bloodstream infections owing to GNB, with a minimum inhibitory concentration of ≤ 0.5 mg/L. Ceftazidime-avibactam is a last-resort antibiotic against GNB of Ambler class A/C/D enzyme-producers and a majority of CR-*P. aeruginosa* isolates. Furthermore, ceftolozane-tazobactam is shown to exhibit excellent *in vitro* activity against CR- and XDR-*P. aeruginosa* isolates. Several pharmaceuticals have devoted to exploring novel antibiotics to combat these troublesome XDR-GNBs. Nevertheless, only few antibiotics are shown to be effective *in vitro* against CR/XDR-*A. baumannii* complex isolates. In this era of antibiotic pipelines, strict implementation of antibiotic stewardship is as important as in-time isolation cohorts in limiting the spread of CR/XDR-GNB and alleviating the worsening trends of resistance.

## Introduction

Infections caused by multidrug-resistant (MDR) and extensively drug-resistant (XDR) Gram-negative bacteria (GNB) have become major challenges for global health institutions because of the limited antibiotic options and high mortality rates (Li et al., 2020; Chen H.Y. et al., 2021; Wang et al., 2021; Zhang et al., 2021b). Among the clinically important XDR-GNB species that harbor plasmidic genes encoding a wide variety of carbapenemases, isolates of sequence type (ST) 258, ST11, and ST147 Enterobacterales (mainly producing *Klebsiella pneumoniae* carbapenemase [KPC], metallo-β-lactamase [MBL, especially New Delhi MBL {NDM}], and oxacillinase [OXA], *etc.*), ST111 and ST235 *Pseudomonas aeruginosa* (mainly producing NDM, imipenemase [IMP], Verona integron-encoded MBL [VIM], and OXA, *etc.*), and ST2, ST32, ST92, and ST368 *Acinetobacter baumannii* complex (mainly producing MBL, NDM, and OXA, *etc.*), they usually co-harbor other resistant β-lactamases (various extended-spectrum β-lactamases [ESBLs] and/or AmpC β-lactamases), thus constitute a worrisome global threat because of their high potential for transmission (Tsakris et al., 2006; Vourli et al., 2006; Miriagou et al., 2007; Zappas et al., 2008; Giakkoupi et al., 2009; Da Silva et al., 2010; Kumarasamy et al., 2010; Papagiannitsis et al., 2010; Wang et al., 2010; Gupta et al., 2011; Chang et al., 2015; Huang et al., 2016; Roy Chowdhury et al., 2017; Yang et al., 2018; Nordmann and Poirel, 2019; Tavoschi et al., 2020; Yoon and Jeong, 2021). Similar to other surveys (Papadimitriou-Olivgeris et al., 2013; Lübbert et al., 2014), the study conducted by McConville et al. revealed that colonization with carbapenem-resistant (CR) Enterobacterales (CRE), which is a prerequisite for CRE infection (Kelly et al., 2017), was an independent predictor of 90-day mortality (adjusted odds ratio [OR]: 2.3; 95% confidence interval [CI]: 1.0–5.3; *P* = 0.056) (McConville et al., 2017). Furthermore, infections caused by CR- or carbapenemase-producing (CP)-GNB were shown to result in high mortality rates (Morata et al., 2012; Poulikakos et al., 2014; Cheng et al., 2015; Micek et al., 2015). This review focuses on global trends, resistance mechanisms, economic burdens, infection control policy and treatment options (including β-lactam combination agents with avibactam, zidebactam, enmetazobactam, relebactam, vaborbactam, nacubactam, durlobactam, and taniborbactam) of CR- and CP-GNB.

## Global Trends of Carbapenem Resistance Among Important GNB Species

**Figure 1:**
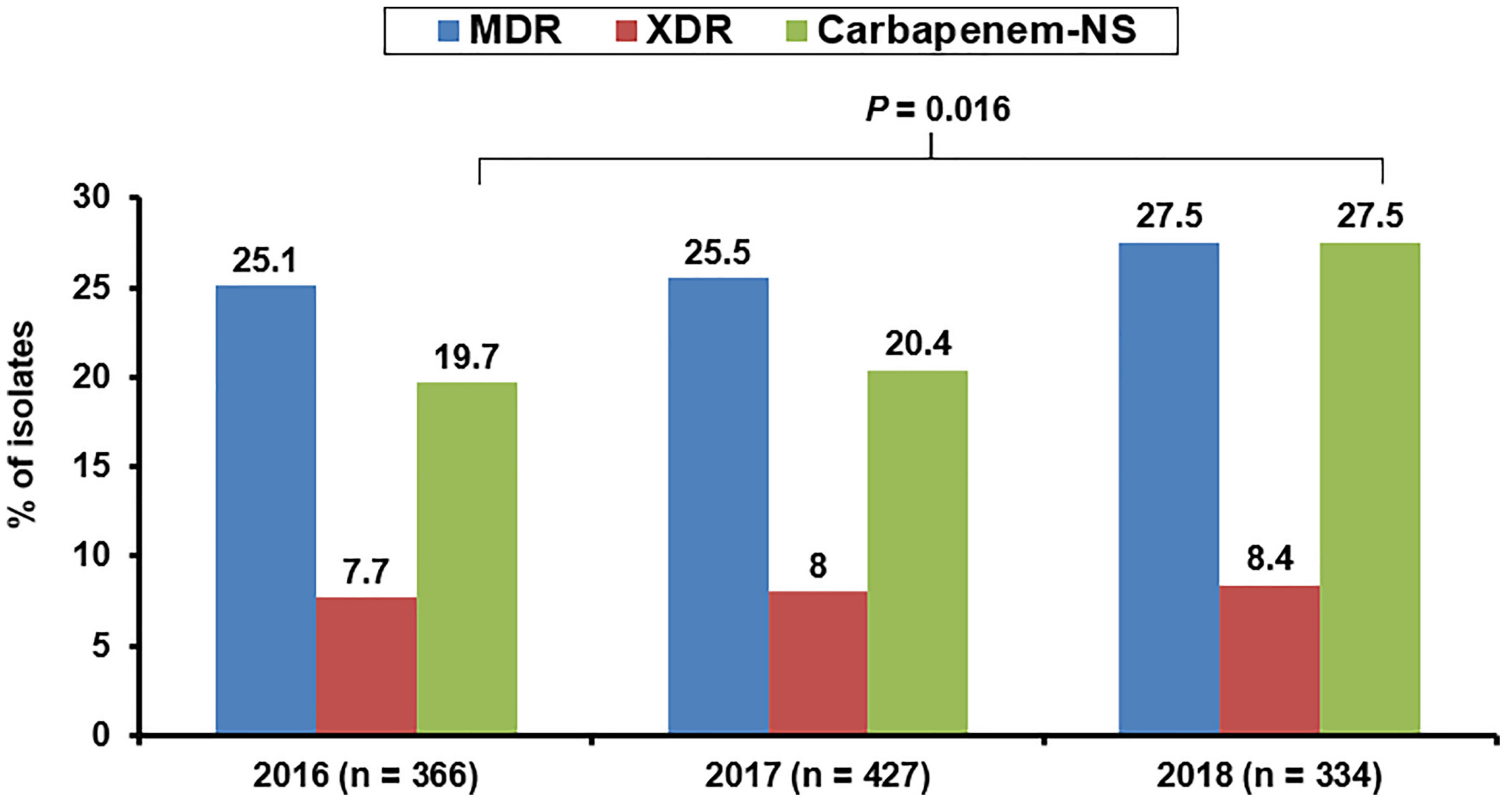
Annual rates of non-susceptibility to any anti-pseudomonal carbapenem agent among *Pseudomonas aeruginosa* cultured from three infection sources (respiratory tract, abdomen, and urinary tract) of patients hospitalized in Taiwan between 2016 and 2018.

The increase in carbapenem resistance in clinically important GNB gradually worsened after 2005, particularly in GNB isolates cultured from patients hospitalized in the intensive care unit (ICU) (Davoudi-Monfared and Khalili, 2018). For example, from 2007 to 2008, Iran reported high imipenem resistance and MDR rates (41.8% and 56.3%, respectively) among *P. aeruginosa* isolates, especially those carrying class 1 integrons, or those cultured from patients undergoing surgery or hospitalized at the burn unit (Yousefi et al., 2010). Additionally, high MDR/CR rates (> 30%) among HAP-related *P. aeruginosa* isolates were observed in many member states of the European Union (EU) in the last decade (Micek et al., 2015). In contrast, the MDR rate of Taiwanese *P. aeruginosa* isolates was less than 18% before 2015. However, the 2016–2018 susceptibility survey of 1,127 *P. aeruginosa* isolates in Taiwan revealed a trend toward prominent escalation in the annual non-susceptible (NS) rates to anti-pseudomonal carbapenems (from 19.7% in 2016 to 27.5% in 2018 [**Figure 1**]; 95% CI: 0.545–0.936; *P* = 0.016) (Jean et al., 2021). When assessed using linear regression analysis, these results corresponded with the annual proportions of pneumonia-causing *P. aeruginosa* isolates (*r* = 0.980, *P* = 0.127) (Jean et al., 2021). An outbreak of XDR-*P. aeruginosa* infections was reported in a tertiary care pediatric hospital in Italy between 2011 and 2012 (Ciofi Degli Atti et al., 2014). The risk of colonization with CP-*P. aeruginosa* isolates harboring *bla*_VIM_ was also reported to apparently increase with the length of hospital stay (especially > 30-day durations) (Neidhöfer et al., 2021).

After 2005, the isolates of MDR-*A. baumannii* complex harboring *bla*_OXA-51_ on the IS*Aba1* element and/or *bla*_VIM_/*bla*_IMP_ on the class 1 integron displayed 45%–80% carbapenem resistance rates and were frequently associated with several clusters in the southern states of the EU after 2005 (Kempf and Rolain, 2012). Moreover, the plasmids on *Acinetobacter* species and some clinical *A. baumannii* isolates that harbor genetic determinants encoding various carbapenem-hydrolyzing class D β-lactamases (*bla*_OXA-23_, *bla*_OXA-58_, *bla*_OXA-58_-like, *bla*_OXA-72_, *etc.*) have also been shown to confer high-level resistance to all carbapenem agents in China and Taiwan (Jean et al., 2015). A study conducted at a German hospital by Neidhöfer et al. observed that the *bla*_OXA-23_-encoding *A. baumannii* complex was more frequently introduced into the hospital by patients residing in the Arabian Peninsula than those of German ethnicity, raising the concern of ethnic factors affecting the infection due to CP-*A. baumannii* (Neidhöfer et al., 2021). Furthermore, nosocomial infections due to MDR-*A. baumannii* complex in pediatrics hospitalized at ICU were reported in Turkey (Ozdemir et al., 2011). In contrast to a few Asian countries where an escalating antimicrobial resistance rate was observed among isolates of *A. baumannii* complex after 2011 (Jean et al., 2013), isolates of *A. baumannii* complex accounted for solely 2.8% of the implicated organisms among episodes of hospital-acquired pneumonia (HAP) acquired in ICUs in US hospitals between 2015 and 2017 (Sader et al., 2018).

In 2012, a 4.6% colonization rate was reported in a rectal swab CRE survey (utilizing MacConkey agar plate for culture and Xpert MDRO cartridge testing) for patients residing in the nursing homes of Rhode Island, USA (Cunha et al., 2016). In stark contrast, Asia and Africa are two leading continents with the highest global CR prevalence rates among Enterobacterales species (Tilahun et al., 2021). Notably, numerous outbreaks related to CRE involving a wide range of microbial species harboring diverse carbapenemase-encoding genes (*bla*_KPC_, *bla*_NDM_, *bla*_VIM_, *bla*_OXA_, *etc.*) have been reported in the literature (Chitnis et al., 2012; Kanamori et al., 2017; Mehta and Muscarella, 2020; Tavoschi et al., 2020; De Man et al., 2021). From March 2017 to September 2018, an epidemic spread of CRE, which was rapidly transmitted between hospitalized patients and associated with high mortality rates at 12 Vietnamese hospitals was reported by Tran et al. (Tran et al., 2019). Hospital-acquired infections and carbapenem therapy were determined to be two independent risk factors contributing to CRE colonization (ORs: 1.74 and 1.79, respectively) (Tran et al., 2019). It is noteworthy that CRE infections were also reported among pediatrics with co-morbidities during the last decade in Los Angeles, USA (Pannaraj et al., 2015). Additionally, endoscopy has surprisingly been found to be an under-recognized source of CRE transmission (Mehta and Muscarella, 2020). Consequently, there is a strong need for the utilization of high-level disinfection for cleaning the endoscopic equipment.

An outbreak owing to genetically related KPC-producing *K. pneumoniae* that persistently existed between March 2009 and February 2011 was reported at one acute care hospital in the USA (Chitnis et al., 2012). In addition, between November 2018 and October 2019, a delayed identification of a cluster involving 1,645 patients who were infected or colonized with NDM-producing CP-Enterobacterales (CPE) was reported in Tuscany, Italy. The majority (accounting for 90.9%) of the implicated CPE isolates that were mostly cultured from the intestinal tract of a total of 1,270 (77.2%) cases were ST147 and *bla*_NDM-1_-harboring CP-*K. pneumoniae* (Tavoschi et al., 2020).

*Stenotrophomonas maltophilia*, intrinsically resistant to all carbapenems, is an environmental MDR organism. Although no dominant *S. maltophilia* clone was identified globally (Kaiser et al., 2009; Duan et al., 2020), it has emerged as an important hospital-acquired pathogen globally (mainly causing primary bacteremia, pneumonia, catheter-associated infection, *etc.*) among hospitalized patients who receive broad-spectrum antibiotics (especially β-lactams) and/or immunosuppressive agents (Falagas et al., 2009; Brooke, 2012).

## Resistance Mechanisms And Case-Fatality Rates Due To Important CR-GNB Infections

Research has revealed that isolates of *A. baumannii* possess several virulence factors, including pili, outer membrane protein A, lipopolysaccharide capsule, and phospholipase (Richards et al., 2015). Resistance mechanisms of MDR or XDR-*A. baumannii* complex, listed as one of the critical priority pathogens by experts of the World Health Organization (Tacconelli et al., 2018), usually include paucity of porins, constitutional expression of efflux pumps (AbeABC, AbeFGH, and AbeIJK), and expression of genes encoding various resistance β-lactamases (AmpC cephalosporinase, class B [NDM and VIM] and/or class D [OXA-23, OXA-58-like, *etc.*] β-lactamases) (Piperaki et al., 2019). High carriage rates of diverse types of OXA enzymes (*bla*_OXA-51_ and *bla*_OXA-23_, followed by *bla*_OXA-58_, *bla*_OXA-24/40_-like, *etc*.)-encoding genes (77%–100%) have been reported in clinical CR-*A. baumannii* isolates globally (Chang et al., 2015; Cortivo et al., 2015; Elabd et al., 2015; Lowings et al., 2015; Kateete et al., 2016; Wang T. H. et al., 2018; Ezadi et al., 2020). Furthermore, several acquired insertion sequences or transposons have been determined to promote the overexpression and spread of plasmid-associated *bla*_OXA-58_ genes in *Acinetobacter* species (Chen et al., 2010; Jean et al., 2015). In contrast to other regions, NDM-encoding genes have been frequently detected in CR-*A. baumannii* isolates in India (Vijayakumar et al., 2016). They frequently caused ventilator-associated pneumonia (VAP), catheter-associated, and bloodstream infections (BSIs) among debilitated patients hospitalized in the ICU, resulting in > 50% case-fatality rates (Poulikakos et al., 2014; Cheng et al., 2015).

Infections caused by CR/MDR-*P aeruginosa* are also usually observed in immunocompromised patients (recipients of chemotherapy and/or high-dose corticosteroids), who suffer from pneumonia (Morata et al., 2012) and require mechanical ventilation (Richards et al., 2015). Micek et al. observed that the prevalence rates of MDR phenotypes among *P. aeruginosa* isolates implicated in HAP in Europe between 2013 and 2014 ranged from 22.2% to 44.2% (Micek et al., 2015). Furthermore, compared to pneumonia due to non-CR/MDR *P. aeruginosa* isolates, patients with pneumonia caused by CR/MDR-*P. aeruginosa* had higher lengths of mechanical ventilation (13.1 days *vs* 17.0 days; *P* = 0.006) (Micek et al., 2015). The case-fatality rates among patients with pneumonia caused by CR/MDR-*P. aeruginosa* have been reported to range from 44.7% to 64% (Morata et al., 2012; Micek et al., 2015).

A study conducted by Kao et al., who analyzed the resistance mechanisms in 87 BSI-causing imipenem-resistant *P. aeruginosa* isolates collected in southern Taiwan between 2000 and 2010, revealed that carbapenemases (mainly VIM and OXA), active efflux pumps, and AmpC β-lactamase overproduction were found in 10.3%, 74.4%, and 51.3% of the *P. aeruginosa* isolates, respectively (Kao et al., 2016). The prevalence rate of metallo-β-lactamase (VIM, 6.4%) among Taiwanese imipenem-resistant *P. aeruginosa* isolates was similar to that in another Chinese study (8.5%) (Wang et al., 2010). However, another molecular study that examined CR and XDR-*P. aeruginosa* isolates (n = 466) collected in Canada between 2007 and 2016 revealed that solely 4.3% (n = 20) harbored a carbapenemase-encoding gene, with Guiana extended-spectrum-5 producers (n = 7 [35%]) surpassing other β-lactamase producers (McCracken et al., 2019). In stark contrast, the 2015–2017 CR-*P. A* study by Schäfer et al. at three medical centers in Germany revealed that 30.6% (19/62) of the samples harbored either *bla*_VIM-1_ (n = 2) or *bla*_VIM-2_ (n = 17) genes (Schäfer et al., 2019). The *bla*_VIM_ carriage rate among CR-*P. aeruginosa* isolates was similar to that in the 2007–2009 survey conducted in Uganda (32%) (Kateete et al., 2016), and that in the 2015–2016 survey of Dubai hospitals in the United Arab Emirates (32%) (Moubareck et al., 2019). Among worldwide MDR-*P. aeruginosa* isolates collected in the last decade, clones ST111, ST175, and ST235 have been identified to carry genomic islands (Roy Chowdhury et al., 2017; Yoon and Jeong, 2021). ST235 (producing KPC, and mainly identified in Europe and Asia) and ST111 (producing VIM-2, and mainly identified in all six continents, except Oceania) clones are the most worrisome class A/B carbapenemase-producers of *P. aeruginosa*, because they are virulent and associated with poor outcomes (Yoon and Jeong, 2021). IMP followed by NDM and VIM have become the two most prevalent class B carbapenemases in worldwide *P. aeruginosa* isolates (Yoon and Jeong, 2021).

In the BSI-CRE (n=83) study conducted by Tamma et al. at the Johns Hopkins Hospital, USA, between March 2013 and April 2016, 37 (45%) isolates were CP-CRE, of which 92% harbored the *bla*_KPC-2_ gene (Tamma et al., 2017). The CP prevalence rate was highly similar to that reported in a Taiwanese 2017 study (Jean et al., 2018a). In contrast, approximately 90% (121/135) of CRE isolates collected from cancer patients between October 2016 and September 2017 in Egypt, where NDM and OXA-48-like enzymes are prevalent, harbored one or more of the carbapenemase-encoding genes, as revealed by polymerase chain reaction (PCR) (Tawfick et al., 2020). Moreover, PCR analysis of rectal swabs from 590 patients hospitalized in Kuwait between April 2017 and March 2018 revealed that 38 (65.5%) out of the 58 patients (9.8%) with rectal CRE colonization harbored the *bla*_OXA-181_-like gene (combined with *bla*_KPC-2_ [n = 5], *bla*_VIM-1_ [n = 4], and *bla*_NDM-5_ [n = 3]) (Fadhli et al., 2020). Previous studies have revealed that high treatment failure rates are associated with KPC-producing Enterobacterales infections, especially among immunocompromised hosts (Jean et al., 2015; Tawfick et al., 2020). In similarity to invasive infections (bacteremia and pneumonia) that are caused by MDR-*P. aeruginosa* or MDR-*A. baumannii* complex resulting in poorer outcomes than those caused by susceptible isolates (Lee et al., 2011; Micek et al., 2015), the all-cause mortality rates related to diverse CRE infections have been notably reported to range from 22% to 72% (Borer et al., 2009; Hirsch and Tam, 2010a; Kalpoe et al., 2012; Tumbarello et al., 2012; Chen H. Y. et al., 2021). Furthermore, a study conducted by Tamma et al. revealed that 32% (12/37) of patients infected with CP-CRE BSIs died within 14 days of admission, with an adjusted OR of 4.92 as compared to non-CP-CRE BSI (Tamma et al., 2017).

The major mechanisms of resistance to carbapenems in *S. maltophilia* isolates mainly include plasmid-encoding L1/L2 β-lactamases (Avison et al., 2001), and chromosomally encoded MDR efflux pumps (Poole, 2004). High rates of case-fatality (42.6%) and attributable mortality (37.5%) were notably reported in patients with *S. maltophilia*-causing pneumonia and septicemia, respectively (Falagas et al., 2009; Tseng et al., 2009). Additionally, a Taiwanese study regarding *S. maltophilia* bacteremia indicated that pediatric patients who had malignancy or failed to remove central venous catheters were at high risk of in-hospital mortality (Wu et al., 2006).

**Table 1** presents the prevalence rates of genes encoding carbapenemases among isolates of CRE, CR-*Pseudomonas aeruginosa*, and CR-*A. baumannii* in different surveys conducted in different countries. Additionally, **Table 2** illustrates the enzymatic and non-enzymatic mechanisms conferring carbapenem resistance among isolates of CRE, CR-*P. aeruginosa*, and CR-*A. baumannii* complex.

**Table 1:** Rates of gene(s) encoding carbapenemases among isolates of carbapenem-resistant (CR) Enterobacterales, CR- *Pseudomonas aeruginosa* and CR-*Acinetobacter baumannii* complex in different surveillances.

Surveillance	Rates (%) of gene(s) encoding carbapenemase(s)	Main carbapenemase(s)	Country	Study period
CR-*P. aeruginosa*				
Wang et al., 2010	8.5	IMP-9, VIM-2	China	2006–2007
Kao et al., 2016	6.4	VIM-3, VIM-2, OXA-10	Taiwan	2000–2010
McCracken et al., 2019	4.3	GES-5, VIM-2, VIM-4	Canada	2007–2016
Schäfer et al., 2019	30.6	VIM-2	German	2015–2017
Kateete et al., 2016	32	VIM	Uganda	2007–2009
Moubareck et al., 2019	32	VIM	United Arab Emirates	2015–2016
CR-Enterobacterales				
Tamma et al., 2017	45	KPC	USA	2013–2016
Jean et al., 2018b	45	KPC-2	Taiwan	2017
Tawfick et al., 2020	90	NDM, OXA-48-like	Egypt	2016–2017
Fadhli et al., 2020	65.5	OXA-181-like	Kuwait	2017–2018
CR-*A. baumannii*				
Chang et al., 2015	80.6	OXA-23-like	China	2012-2013
Kateete et al., 2016	93.3	OXA-23-like, OXA-58-like, VIM-like	Uganda	2007-2009
Cortivo et al., 2015	87.3	OXA-23-like, OXA-51-like	Brazil	2010-2013
Elabd et al., 2015	94.7	OXA-23-like, OXA-40-like, OXA-58-like	Saudi Arabia	2013-2014
Ezadi et al., 2020	100	OXA-23-like, OXA-24/40-like	Iran	2016-2017

CR, carbapenem-resistant; KPC, Klebsiella pneumoniae carbapenemase; VIM, Verona integron-encoded metallo-β-lactamase; NDM, New Delhi metallo-β-lactamase; OXA, oxacillinase; IMP, imipenemase; GES-5, Guiana extended-spectrum-5 carbapenemase.

**Table 2:** The mechanisms of carbapenem resistance in carbapenem-resistant (CR) Enterobacterales species, CR-*Pseudomonas aeruginosa*, and CR-*Acinetobacter baumannii* complex.

Species	Main mechanisms of resistance to carbapenems	
	Enzyme-mediated	Non-enzyme-mediated
CR-Enterobacterales	Various carbapenemases (Ambler class A, B, D) ± ESBL or AmpC β-lactamase(s)	Porin (OmpK35, OmpK36) loss (plus ESBL or AmpC β-lactamase [resistant to ertapenem, imipenem])
		Multidrug-resistant efflux pump (e.g., AcrAB-TolC system)
CR-*Pseudomonas aeruginosa*	Carbapenemases (Ambler class B β-lactamases predominantly)	Porin (OprD) loss (plus hyper-production of Ambler class C enzyme)
	Pseudomonas-derived cephalosporinase (PDC, Ambler class C)	Multidrug-resistant (tripartite) efflux pump
CR-*Acinetobacter baumannii* complex	Carbapenemases (Ambler class B and D predominantly)	Porin loss
	Ambler class C cephalosporinase hyper-production	Multidrug-resistant efflux pump (e.g., AdeABC, encoded by *adeB*, *adeG*, and *adeJ*, *etc.*)

ESBL, extended-spectrum β-lactamase.

## Economic Burden Caused by CRE Outbreaks And Screening Strategies

The huge economic burden caused by MDR-GNB infections, most of which were limited to CRE, has been intensively analyzed. Bartsch et al. investigated the median cost of CRE infections with an incidence of 2.93 per 100,000 persons in the USA in 2015. They estimated that it cost hospitals 275 million US dollars, third-party payers 147 million US dollars, and society 553 million US dollars. In addition, costs increased proportionally with the incidence of CRE, with increases of 2.0-fold, 3.4-fold, and 5.1-fold for incidence rates of 6, 10, and 15 per 100,000 persons, respectively (Bartsch et al., 2017). In-hospital outbreaks of CR- or CP-GNB further exacerbate the already high-cost burden. An outbreak of CPE (caused by mainly NDM-producing *K. pneumoniae*), which occurred at a London hospital from July 2014 to October 2015, cost €1.1 million, with €54,000 spent on antibiotics for 18 patients who needed treatment, €94,000 on laboratory screening, €153,000 on Estates renovations, and €822,000 as opportunity cost (staff time, bed closure, and elective surgical missed revenue) (Otter et al., 2017). Furthermore, an investigation conducted by Yang et al. at a tertiary teaching hospital in China from 2011 to 2016 revealed that the direct economic burden and disability-adjusted life-year loss caused by BSI due to CR-*P. aeruginosa* was ≥ 3-fold higher than that of carbapenem-susceptible *P. aeruginosa* BSI (Yang et al., 2021). According to Semin-Pelletier et al., frequent readmissions of patients who had previous infections due to OXA-48-producing *K. pneumoniae* isolates, the large number of transfers between wards, and a delay in the implementation of successive cohort units greatly contributed to the incomplete success of containing the spread of CPE (Semin-Pelletier et al., 2015). A few physicians have adopted aggressive surveys of samples from patients to interrupt the transmission of CRE/CPE. For example, a survey at a tertiary teaching hospital in Malaysia revealed that 5.74% of Enterobacterales isolates cultured from various clinical samples (of which rectal swab screening accounted for 49.3%) were CRE, most of which were *K. pneumoniae* isolates harboring *bla*_NDM-1_ (Zaidah et al., 2017). In the multicenter survey conducted by Jimenez et al. in Miami, Florida from 2012 to 2016, active surveillance testing revealed that the overall CRE prevalence was 0.077 cases per 100 patient-admissions, while the incidence density was 1.46 cases per 10,000 patient-days. It is also noteworthy that rates of CRE (dominated by *K. pneumoniae*, *Enterobacter cloacae*, and *Escherichia coli*) steadily increased during the first 3 years of the study period, and declined after implementation of infection control strategies (contact precautions, environmental disinfection, *etc.*) (Jimenez et al., 2020).

Owing to the high burden of medical costs and high case-fatality rates seen for inpatients with CPE infections, attempts have been made to improve the cost-effectiveness of screening for CPE among all hospital inpatients (Kang et al., 2020). Among the different culture methods, the ChromID CARBA method (bioMérieux, Marcy l’Etoile, France) has been to perform rapidly and best in the detection of CPE (> 100 colony forming units/spot) from rectal swabs, in terms of sensitivity (92.4%) and specificity (96.9%) (Vrioni et al., 2012; Wilkinson et al., 2012). Despite a lower reagent cost, culture-based methods are, in fact, less sensitive than molecular methods for the detection of infection or colonization of CPE. Moreover, they are labor-intensive and slower to yield results (Richter and Marchaim, 2017). Lapointe-Shaw et al. observed that screening for CPE might be cost-effective as compared to not screening, if the prevalence of CPE was above 0.3% among the isolates of Enterobacterales under survey (Lapointe-Shaw et al., 2017).

## Community-Acquired/Community-Onset CRE, and CRE, CR-*P. Aeruginosa* as Well as CR-*A. Baumannii* Complex Recovered from The Long-Term Care Facility (LTCF)

Apart from hospital acquired CRE, the presence of CRE in the community setting is also a potentially worrisome threat to the public health of ambulatory patients (Van Duin and Paterson, 2016; Jean et al., 2018b). A meta-analysis conducted by Kelly et al. revealed that 5.6%–10.8% of rates of colonized CRE isolates belonged to the community-associated/community-onset category (CA/CO; defined as identification of asymptomatic CRE colonization at the time of admission) in the USA-based studies, while percentages ranging from 0.04% to 29.5% of colonized CRE categorized as CA/CO in origin were reported globally (Kelly et al., 2017). Compared to healthcare-acquired CRE, a Taiwanese survey conducted by Tang et al. revealed that CA-CRE was more likely to occur in elderly female patients and result in urinary tract infection (UTI) (Tang et al., 2016). In a study conducted in China, Hu et al. also found that CO-CRE isolates (n = 28, accounting for 43.8% of all infection-causing CRE isolates enrolled) were mainly cultured from urine samples (75%). Of the CO-CRE isolates, 8 (28.6%) were clonally unrelated *E. coli* isolates, all of which were NDM producers that were less resistant to aztreonam, ciprofloxacin, levofloxacin, and chloramphenicol*. pneumoniae* (Hu et al., 2020).

The emergence of CRE at an LTCF (an institution that provides long-term rehabilitation and skilled nursing care) is also a major healthcare issue (Vrioni et al., 2012). The prevalence and incidence rate of colonized CRE in LTCFs in the USA ranged from 3% to 30.4% (Lin et al., 2013; McKinnell et al., 2019) and 1.07 to 6.83 cases per 10,000 patient-days (Brennan et al., 2014; Chopra et al., 2018), respectively. In contrast, the CRE prevalence rates differed widely among the states of the EU (Chen C.C. et al., 2021). A significant association (ranging from 12% to 15.5%) between CRE colonization in nursing home residents with hospital admissions was observed in Spain (predominantly *K. pneumoniae* harboring *bla*_OXA-48_-like gene) (Palacios-Baena et al., 2016) and Israel (mainly KPC-producing *K. pneumoniae*) (Ben-David et al., 2011). Accordingly, nursing home residents have become CRE reservoirs that should not be ignored. Multiple risk factors, including fecal incontinence (OR: 5.78) (Mills et al., 2016), an immunocompromised condition (OR: 3.92), comorbidities (Charlson score > 3; OR: 4.85) (Bhargava et al., 2014), use of gastrointestinal devices (OR: 19.7) (McKinnell et al., 2019), intravascular indwelling devices or urinary catheters (OR: 5.21) (Lin et al., 2013), and mechanical ventilation (OR: 3.56) (Mills et al., 2016), sharing a room with a known CRE carrier (Chitnis et al., 2012), and prior antibiotic exposure (OR: 3.89) (Chitnis et al., 2012; Bhargava et al., 2014; Brennan et al., 2014), *etc.*, has significantly associated with increased vulnerability to CRE colonization and/or infections in LTCF residents. It is noteworthy that patients from LTCFs who were colonized or infected with CRE had notably poor clinical outcomes, with a mortality rate of up to 75% among patients with CRE infection (Borer et al., 2012). In contrast, the prevalence rates of CRE colonization among residents of LTCFs ranged from 13% to 22.7% in Asia (Lee et al., 2017; Hagiya et al., 2018; Jean et al., 2018b; Le et al., 2020). As stated in a survey on the distribution of carbapenemase-encoding genes among LTCF residents (Jean et al., 2015), ST258 CR-*K. pneumoniae* harboring *bla*_KPC-2_ or *bla*_KPC-3_ were the predominant clones present in residents of LTCFs in the USA, whereas other carbapenemases (including NDM, VIM, IMP, and OXA-48-like) were uncommon among CRE isolates for them (Prasad et al., 2016; Reuben et al., 2017; Dubendris et al., 2020; Chen C.C. et al., 2021). In Japan, various types of IMP (predominantly IMP-42, IMP-11, followed by IMP-6) carbapenemases have been detected among overall CRE isolates in LTCF residents in Japan (Hagiya et al., 2018; Hayakawa et al., 2020). Data from the CRE survey of LTCF residents in Spain revealed that OXA-48-like enzymes, followed by VIM-1, IMP, and KPC are the main carbapenemases that can be found there (Palacios-Baena et al., 2016).

Although *P. aeruginosa* is not a common colonized GNB cultured from LTCF residents, as reported by the survey conducted by O’Fallon et al. in the USA (O’Fallon et al., 2009), and Italian surveys of Giufrè et al. (Giufrè et al., 2017) and March et al. (March et al., 2017), it has been shown to be easily transmitted through numerous routes, including patient-to-patient contact and environmental contamination (Jefferies et al., 2012). Thus, it is plausible that the CP-*P. aeruginosa* (harboring *bla*_VIM_) isolates were cultured from LTCF residents who have history of frequent hospitalizations (Nucleo et al., 2018). A study conducted by Raman et al. revealed that MDR/CR-*P. aeruginosa* can be identified from patients who were transferred from chronic care facilities and exposed to piperacillin-tazobactam (adjusted OR: 2.64) or carbapenem (adjusted OR: 4.36) (Raman et al., 2018). 

**Table 3** illustrates the summary of CRE, CR-*P. aeruginosa*, and CR-*A. baumannii* cultured from the community setting and LTCF.

**Table 3:** Summary of reports on carbapenem-resistant (CR) Enterobacterales (CRE), and CRE, CR-*P. aeruginosa*, and CR-*A. baumannii complex* at the community setting, community-acquired/community-onset (CA/CO), and the long-term care facility (LTCF).

CR-GNB species, settings, and surveillances	Rates (%) of gene(s) encoding carbapenemase(s)	Main carbapenemase(s)	Country	Study period
CR-Enterobacterales, **CA/CO**				
Kelly et al., 2017	5.6-10.8	KPCs	USA	2008-2013
Tang et al., 2016	29.5	NDM, KPC-2	Taiwan	2015
Hu et al., 2020	43.8	KPC-2	China	2015-2018
CR-Enterobacterales, **LTCF**				
Lin et al., 2013	30.4	KPC	USA	2010-2011
McKinnell et al., 2019	3	KPC	USA	2016-2017
Palacios-Baena et al., 2016	15.5	OXA-48-like	Spain	2013
Ben-David et al., 2011	12	KPC	Israel	2008
Lee et al., 2017	22.7	KPC-2	Taiwan	2015
Hagiya et al., 2018	13	KPC-2	Japan	2017-2018
Prasad et al., 2016	18.9	KPC-2, KPC-3	USA	NA
Reuben et al., 2017	5.2	KPC-2	USA	2016
Dubendris et al., 2020	4.8	KPC-2	USA	2016-2017
Hagiya et al., 2018	19.3	IMP	Japan	2015-2016
Hayakawa et al., 2020	30	IMP	Japan	2016-2018
CR-*P. aeruginosa*, **LTCF**				
Giufrè et al., 2017	NA	NA	Italy	2015
March et al., 2017	NA	NA	Italy	2008, 2012
Nucleo et al., 2018	NA	GES-5	Italy	2016
CR-*A. baumannii*, **LTCF**				
Sengstock et al., 2010	NA	NA	USA	2003-2008
Mody et al., 2015	NA	NA	USA	NA

NA, non-applicable; KPC, Klebsiella pneumoniae carbapenemase; NDM, New Delhi metallo-β-lactamase; OXA, oxacillinase; IMP, imipenemase; GES-5, Guiana extended-spectrum-5 carbapenemase.

Bold values (CA/CO, LTCF) mean the culture settings of CR organisms.

## Infection Control Policy Regarding CR- or XDR-GNB

Early identification of in-hospital patients with risk factors for CRE acquisition (72.7% of which were *K. pneumoniae*), such as co-colonization with MDR-*A. baumannii* complex (adjusted OR: 15.6) or ESBL-producing GNB (adjusted OR: 4.7; might be linked to recent carbapenem use), exposure to glycopeptide antibiotics (adjusted OR: 3.6), and admissions within one year (adjusted OR: 3.9), is beneficial for avoiding potential spread (Kang et al., 2019). In addition, excessive consumption of carbapenems (especially group 2 agents) is considered to be an important predisposing factor that contributes to the worsening rates of infections caused by CR-*P. aeruginosa* (estimated OR: 2.87 - 40.96) (Voor In ’t Holt et al., 2014), CRE (McLaughlin et al., 2013), and CR-*A. baumannii* complex (Sheng et al., 2010; Yoon et al., 2014). Consequently, appropriate antibiotic control policies (especially strict implementation of antibiotic stewardship) in hospitals, education of primary care staff to prevent the dissemination of high-risk hospital and LTCF microorganisms, and contacting isolation cohorts (Barlam et al., 2016; Hellyer et al., 2016) are of paramount importance in containing the spread of CR- or MDR/XDR-GNB strains and decreasing case-fatality rates. Similarly, there is a need to implement periodical surveillance testing to determine in-time targeted interventions (e.g., isolating or cohorting CRE carriers and nursing staff) are needed to effectively lessen the CRE outbreaks at LTCFs (Ben-David et al., 2011; Chitnis et al., 2012; Chen H. Y. et al., 2021).

## Different Phenotypic, Biochemial and Immunological Techniques for Diagnosis of CR-GNB

The distinguishment of CR-GNB that are due to the non-carbapenemase-mediated mechanisms from CP-GNB is important, because CP-GNB are prone to disseminate between patients more readily than non-CP-GNB (Goodman et al., 2016). Among the phenotypic CRE diagnostic assays commonly used in clinical microbiology laboratories, the modified Hodge test was applied earliest to detect potential KPC producers of CRE. However, it showed poor sensitivity in detection of the NDM and OXA-48-like CPE, and false-positive results are seen in CRE owing to the porin alteration combined with hyperproduction of ESBL and/or AmpC β-lactamase (Tamma and Simner, 2018; Clinical and Laboratory Standards Institute (CLSI), 2021). The Carba NP test and its variants are suitable for detection of various carbapenemases in CRE and CR-*P. aeruginosa* (Tamma and Simner, 2018). In spite of requiring the acquisition of dedicated reagents and being interpreted subjectively, the Carba NP test is a convenient biochemical test that provides the results within 15-30 minutes and thus has been applied in the clinical microbiological laboratories worldwide (Clinical and Laboratory Standards Institute (CLSI), 2021). Additionally, the CarbAcineto NP test was shown to perform well in detecting carbapenemases produced by CR-*A. baumannii* (Dortet et al., 2014).

Utilization of the modified carbapenem inactivation method (mCIM) in combination with the EDTA-modified CIM (eCIM) test could reliably differentiate MBL-producing CRE strains (those displaying a negative result on only the eCIM test) from serine-class carbapenemase producers (showing positive results for both tests) (Tamma and Simner, 2018; Jean et al., 2019). Nevertheless, it takes approximately 6-12 h to obtain the results and is interpreted subjectively as well. It is also noteworthy that the mCIM/eCIM test performed less well in detection of VIM-producing *P. aeruginosa* and OXA-producing *A. baumannii* isolates than CPE (Tamma and Simner, 2018).

The other carbapenemase detection tests include lateral flow immunoassays (antibody-based rapid diagnostic, easy-to-use methods, such as NG-Test Carba 5 [Hardy Diagnostics, CA, USA], and Resist-3 O.K.N. that detects OXA-48-like, KPC and NDM but not VIM and IMP-like MBLs [Coris BioConcept, Gembloux, Belgium]), targeted carbapenemase assays (inhibitor [phenylboronic acid for KPC and EDTA for MBL]-based, easy-to-use methods) for diverse carbapenemases, and matrix-assisted laser desorption–ionization time of flight mass spectrometry (hydrolysis approach) (Wareham and Abdul Momin, 2017; Tamma and Simner, 2018).

## Antibiotic Treatment Against CR- or XDR-GNB

### Conventional Antibiotic Regimens Against CRE

In a study by Garonzik et al., optimum dosages of polymyxin B and colistin (polymyxin E), two old antibiotics of revival in this century, have been proposed to maximize their efficacy against infections related to MDR-GNB, with a minimum inhibitory concentration (MIC) of >1 mg/L for colistin (Garonzik et al., 2011). Clinical, pharmacokinetic (PK) and pharmacodynamic (PD) data of polymyxin B/E for several GNB species have been shown to have limited clinical efficacy, even if an intermediate result (*i.e.*, MIC ≤ 2 mg/L) was achieved (Clinical and Laboratory Standards Institute (CLSI), 2021). Moreover, monotherapy with intravenous colistin against CR-*K. pneumoniae* or *E. coli* bacteremia was also not suggested, because of its association with a high (57.1%) mortality rate (hazard ratio: 5.57; 95% CI, 2.13 - 14.61; *P* < 0.001) as compared to other comparative antibiotics (Lin et al., 2018). Despite controversies, during the interval of an aerosolized colistimethate sodium (CMS) dosing (2 million units [MU]) using jet or ultrasonic nebulizer, a high pulmonary area under the concentration-time curve of colistin (ranging 18.9 – 73.1 μg•h/mL), as well as a high maximum pulmonary colistin concentration (6.00 ± 3.45 μg/mL) were achieved in humans, with no increase in nephrotoxicity (Yapa et al., 2014). Thus, a high-dose regimen of aerosolized CMS monotherapy (4 MU administered every 8 h) was applied for the treatment of VAP caused by a few notable MDR-GNB (*P. aeruginosa* and *A. baumannii* complex predominantly) (Abdellatif et al., 2016). In addition, impaired uptake of fosfomycin (related to *glpT*, *uhpT*, and *uhpA*) (Takahata et al., 2010) and the existence of fosfomycin-modified genes (e.g., fosA3, which encodes fosfomycin-modifying enzymes) in transposon elements and conjugative plasmids confer Enterobacterales species, especially *E. coli*, exhibiting resistance to fosfomycin (Yang et al., 2019). High prevalence rates of fosfomycin resistance genes in CRE have been determined in several parts of East Asia (e.g., China and Japan) (Zhang et al., 2017; Wang Q. et al., 2018), and resistance to fosfomycin has also emerged in the USA and the states of the EU (Alrowais et al., 2015; Benzerara et al., 2017). Therefore, the use of systemic colistin and fosfomycin is recommended as an adjunctive treatment for CRE or CR-*P. aeruginosa* infections (Amladi et al., 2019; Loose et al., 2019).

Before the launch of ceftazidime-avibactam for the clinical treatment of CPE infection in 2015, Gutiérrez-Gutiérrez et al. concluded that therapy with a combination regimen of antibiotics (including at least one *in vitro* active drug against the implicated BSI isolate and started in the first five days after infection) was associated with decreased 30-day all-cause mortality rates among patients infected with BSI owing to CPE (mainly KPC-producing *K. pneumoniae*) and high INCREMENT-CPE mortality scores (high Pitt bacteremic scores in patients who had ≥ 2 points of Charlson comorbidity score and BSI not originating from UTI or biliary tract infection) (Gutiérrez-Gutiérrez et al., 2016; Gutiérrez-Gutiérrez et al., 2017). Despite its bacteriostatic nature and relatively low serum concentration under standard-dose administration (100 mg loading followed by 50 mg every 12 h) (Jean and Hsueh, 2014), the study by Lin et al. on CRE bacteremia revealed that tigecycline was a good choice if the antibiotic had an MIC ≤ 0.5 mg/L against the isolates, based on the 90% probability of target attainment and 82% probability of cumulative fraction of response (Lin et al., 2018). Nevertheless, heterogeneity in adequate regimens of antibiotics against CPE has been suggested for both *in vitro* efficacy and clinical treatment effects (Bulik and Nicolau, 2011; Jernigan et al., 2012; Tzouvelekis et al., 2014; Lowman and Schleicher, 2015; De Pascale et al., 2017; Tang et al., 2019). For example, Tang et al. determined that *in vitro* treatment with 1× MIC using combinations of amikacin or gentamicin, and tigecycline or doxycycline for 24 h resulted in bactericidal activity of 84%–100% in 13 KPC-*K. pneumoniae* isolates; in addition, the combination of doxycycline plus gentamicin or amikacin was synergistic for all the tested KPC-*K. pneumoniae* isolates (Tang et al., 2019). A review of CPE therapy by Tzouvelekis et al. and an investigation conducted by Daikos et al. concluded that the lowest mortality rate (18.8%) was observed in patients treated with combinations of various antibiotics (Daikos et al., 2014; Tzouvelekis et al., 2014). Compared to other antibiotic regimens, Tumbarello et al. further demonstrated that post-antibiogram therapy with a combination of tigecycline, colistin, and meropenem (at a dosage of 2 g every 8 h intravenously) was associated with lower mortality rates (OR, 0.11; 95% CI: 0.02 - 0.69; and *P* = 0.01) for patients with BSI due to KPC-producing *K. pneumoniae*, especially those with an MIC ≤ 16 mg/L for meropenem (Tumbarello et al., 2012). In addition, Lowman et al. observed that carbapenem-based therapy improved survival in 20 critically ill patients infected with CPE harboring *bla*_OXA-48_-like gene, although the severity of the underlying illness significantly impacted their outcomes as well (Lowman and Schleicher, 2015). The combination of doripenem (8 mg/L) and colistin (1 mg/L) was observed to have *in vitro* bactericidal efficacy against 75% (9/12) of KPC-producing *K. pneumoniae* isolates (Jernigan et al., 2012). As compared to the clinical treatment using a single antibiotic (colistin, tigecycline, or gentamicin), De Pascale et al. determined that a double carbapenem regimen (comprising meropenem at a dosage of 2 g every 8 h intravenously, and ertapenem at a dosage of either 2 g once daily or 1 g every 12 h intravenously) resulted in a significantly lower rate of 28-day mortality (47.9% *vs* 29.2%; *P* = 0.04), higher rates of clinical cure (31.3% *vs* 65%; *P* = 0.03) and microbiological eradication (25.9% *vs* 57.9%; *P* = 0.04) for patients with severe infections due to CR-*K. pneumoniae* (> 90% of which harbor *bla*_KPC_) (De Pascale et al., 2017). A similar *in vivo* effect was also reported by Builk et al. in a murine thigh model with KPC-producing *K. pneumoniae* infection, using simulated mega-dose doripenem (2 g every 8 h) combined with ertapenem (1 g once daily) (Bulik and Nicolau, 2011).

### Novel Antibiotics Against CR/CP-Enterobacterales, and CR-*P. Aeruginosa*

Ceftazidime combined with avibactam, a diazabicyclooctane (DBO) β-lactamase inhibitor, is effective against most Ambler class A/C/D enzymes, providing a new option for the treatment of CRE/CPE infections (Liu et al., 2021); however, mutations in Asp179Tyr, Val240Gly, Ala240Val, Ala177Glu, Thr243Met substitutions, and 165-166Glu-Leu insertion, have been shown to compromise the *in vitro* efficacy of ceftazidime-avibactam while sparing several other novel antibiotics against CRE isolates (Wang Y. et al., 2020). In addition, recent investigations have revealed that a significant proportion of CR-*E. cloacae* complex exhibited significantly higher NS rates to ceftazidime-avibactam (Yin et al., 2019; Kawai et al., 2020). In the small case series covering diverse CRE infections (n = 37, with five-sixths being CR-*K. pneumoniae*), Shields et al. observed that ceftazidime-avibactam therapy achieved a success rate of 70% and 50%, respectively, for bacteremia and pneumonia, and 100% for acute pyelonephritis. Furthermore, resistance to ceftazidime-avibactam among CRE developed notably following its therapy for 10–19 days (median, 15 days) (Shields et al., 2016). The overall 30-day survival rate of the series by Shields et al. was 76% (Shields et al., 2016), similar to that of another survey on CR-*K. pneumoniae* therapy using antibiotic combination regimens (72.5%) (Capone et al., 2013). In a small cohort (n = 38) survey conducted by Alraddadi et al. to explore the outcomes of patients with various infections caused by CRE (largely OXA-48-producing *K. pneumoniae* isolates), ceftazidime-avibactam therapy provided a better clinical remission rate than comparative antibiotics, including colistin, tigecycline, and meropenem (80% *vs* 53.6%; *P* = 0.14) (Alraddadi et al., 2019). Nevertheless, no difference was observed in the 30-day all-cause mortality rates between the two groups (50% *vs* 57.1%; *P* = 0.71) (Alraddadi et al., 2019). Furthermore, in a head-to-head study conducted by Ackley et al. who compared the clinical efficacy and development of resistance between ceftazidime-avibactam and meropenem-vaborbactam (a β-lactamase inhibitor that comprises structurally boronic acid [BA]) against CRE, a 2.9% rate of resistance developed after ceftazidime-avibactam monotherapy, while similar rates of clinical success and 90-day mortality were observed between the two groups (Ackley et al., 2020).

Among other potential novel antibiotics, ceftolozane-tazobactam was inactive against isolates of CPE isolates, several AmpC-producing Enterobacterales, and most ESBL-producing *K. pneumoniae* (Jean et al., 2021). Cefepime in combination with enmetazobactam (formerly AAI101, a β-lactamase inhibitor that structurally comprises penicillanic acid sulfone), exhibited lower MICs against isolates of ESBL-producing Enterobacterales and KPC-producing *E. coli* than CP-*K. pneumoniae* (Morrissey et al., 2019; Papp-Wallace et al., 2019; Jean et al., 2022). In contrast, cefepime combined with zidebactam (formerly WCK 5222, a structural DBO β-lactamase inhibitor) displayed superior *in vitro* activity against the Ambler class B enzyme producers of Enterobacterales than ceftazidime-avibactam and imipenem in combination with relebactam (also a structural DBO β-lactamase inhibitor), meropenem in combination with vaborbactam, cefepime-enmetazobactam, durlobactam (formerly ETX 2514, a structural DBO β-lactamase inhibitor, penicillin-binding protein [PBP] 2 inhibitor, and *in vitro* activity against *P. aeruginosa* AmpC β-lactamase), and plazomycin (Landman et al., 2010; Karlowsky et al., 2020; Novelli et al., 2020; Hagihara et al., 2021; Kuo et al., 2021; Shapiro et al., 2021; Jean et al., 2022). Furthermore, the anti-CPE spectra of aztreonam-avibactam and cefepime combined with taniborbactam [also a structural BA β-lactamase inhibitor against all metallo-β-lactamases (MBL), except for the IMP types], are considerably similar to those of cefepime-zidebactam (Karlowsky et al., 2017; Wang X. et al., 2020; Sader et al., 2021) and cefiderocol (Hsueh et al., 2019). Nacubactam (formerly OP0595 or RG6080, a structural DBO β-lactamase and PBP2 inhibitor) was demonstrated to be active *in vitro* against diverse β-lactamase producers, including a few NDM or VIM-producing Enterobacterales species (mainly *E. coli* and *Enterobacter* spp.) (Mushtaq et al., 2019). When nacubactam was combined with meropenem, it was shown to be active *in vitro* against the CRE isolates of any β-lactamase producer. Phase 3 clinical investigation is currently being conducted to test the efficacy of nacubactam in combination with meropenem against CRE isolates harboring the *bla*_KPC_ gene (Barnes et al., 2019).

As stated in several studies, septicemia or pneumonia caused by MDR-*P. aeruginosa* isolates resulted in poor patient outcomes (Hirsch and Tam, 2010b; Morata et al., 2012; Micek et al., 2015; Matos et al., 2018; Urzedo et al., 2020). Recommendations for the treatment of MDR/XDR-GNB infections have been published based on the susceptibility and PK/PD profiles of conventional and novel antibiotics (Bassetti et al., 2019; Jean et al., 2019; Jean et al., 2020). Antibiotic combination therapy is likely to select mutants displaying a broader resistance phenotype (e.g., mutational inactivation of the repressor gene *mexR* that regulates the multidrug efflux operon *mexAB–oprM* for *P. aeruginosa*) than before (Vestergaard et al., 2016). Nevertheless, a few combination regimens of dual antibiotics exhibited synergistic or additive effects *in vitro* (determined using fractional inhibitory concentration index) against CR- or MDR/XDR-*P. aeruginosa* isolates (Erdem et al., 2002; Siriyong et al., 2019; Olsson et al., 2020). In addition, the combination of gentamicin and ciprofloxacin has good potential for inhibiting biofilm formation synthesized from *P. aeruginosa* (71.4%) (Wang et al., 2019). De-escalation of antibiotics into a single agent is strongly recommended when susceptibility of the implicated GNB (including MDR/XDR-*P. aeruginosa*) isolate is known, and there is a significant improvement in the patient’s condition (Boyd and Nailor, 2011). Among the novel antibiotics that have been launched to combat MDR-GNB isolates, ceftolozane-tazobactam has excellent *in vitro* activity against global CR- and XDR-*P. aeruginosa* strains, including those with overexpression of efflux pumps but no carbapenemase production (Hong et al., 2013; Jean et al., 2021). Despite not being validated by several randomized clinical studies, ceftazidime-avibactam (Sader et al., 2017b; Kuo et al., 2021), cefepime-zidebactam (Sader et al., 2017a; Kuo et al., 2021; Jean et al., 2022), cefiderocol (Hsueh et al., 2019; Yamano, 2019; Bassetti et al., 2021; Liu et al., 2021), imipenem-relebactam (showing excellent *in vitro* activity relative to imipenem solely against OprD-losing *P. aeruginosa* isolates with *Pseudomonas*-derived cephalosporinase hyper-production) (Tselepis et al., 2020; Kuo et al., 2021), meropenem-vaborbactam (Novelli et al., 2020; Kuo et al., 2021), meropenem-nacubactam (Asempa et al., 2020), and cefepime-taniborbactam (Wang X. et al., 2020) are considered as promising alternatives against infections caused by CR- or MDR-*P. aeruginosa* isolates. Although aztreonam-avibactam has excellent *in vitro* potential to inhibit CPE (including producers of MBL; MIC_90_ ≤ 8 mg/L) (Karlowsky et al., 2017; Wang Y. et al., 2020), relatively high MIC_90_ levels of this novel antibiotic were observed against the global overall and MBL-positive *P. aeruginosa* isolates tested (32 mg/L and 32 mg/L, respectively) (Karlowsky et al., 2017).

### Conventional and Novel Antibiotics Against CR-*A. Baumannii*

A 3-h intravenous infusion of 2 g meropenem every 8 h produced a high percentage (72.89 ± 22.40%) of time above the serum concentration of 8 mg/L after the third dose (Jaruratanasirikul et al., 2005). This regimen provides therapeutic benefits for the treatment of VAP caused by CR-*A. baumannii* isolates. Unfortunately, a high *in vitro* resistance to meropenem (MIC > 64 mg/L) was exhibited by most XDR/CR-*A. baumannii* and CRE isolates, whose susceptibility was not restored by sulbactam addition (Hsueh et al., 2002). Because of the extremely high likelihood of resistance to the majority of antibiotics, various regimens of antibiotic combinations have been proposed for the treatment of XDR-*A. baumannii* in the PubMed database. For instance, Jean et al. demonstrated that a prolonged intravenous infusion (> 3 h) of imipenem combined with tigecycline provided a significantly better survival rate for patients with XDR-*A. baumannii* bacteremic VAP than imipenem plus sulbactam (64.3% *vs* 14.3%) (Jean et al., 2016). Time-kill kinetic analysis proved that the former regimen inhibited the *in vitro* growth of XDR-*A. baumannii* (Poulikakos et al., 2014). In addition, treatment with a colistin-carbapenem (doripenem) regimen also significantly improved the 28-day survival rates among solid-organ transplant recipients with various XDR-*A. baumannii* infections (OR: 7.88; 95% CI: 1.60 – 38.76; *P* = 0.01) (Shields et al., 2012). In partial similarity to the meta-analysis conducted by Kengkla et al., who substituted tigecycline for carbapenem (Kengkla et al., 2018), Pongpech et al. suggested that the triple combination of meropenem, colistin, and sulbactam was a good regimen *in vitro* against carbapenem-NS *A. baumannii* complex that did not harbor genes encoding class B carbapenemase (Pongpech et al., 2010). Furthermore, to effectively treat severe VAP or BSI caused by MDR or XDR-*A. baumannii* complex, Piperaki et al. suggested a combination regimen comprising two active *in vitro* agents. Antibiotic options include high-dose ampicillin-sulbactam, high-dose tigecycline (or minocycline), and polymyxin or aminoglycoside. If two *in vitro* active agents are not available, *in vitro* synergy studies are valuable in choosing the most appropriate targeted combination scheme (Piperaki et al., 2019). Novel antibiotics, including ceftolozane-tazobactam, imipenem-relebactam, meropenem-vaborbactam, ceftazidime-avibactam, aztreonam-avibactam, cefepime-enmetazobactam, and cefepime-zidebactam, have significantly poor *in vitro* activity against XDR-*A. baumannii* complex, while exhibiting excellent *in vitro* efficacy against CRE or CR-*P. aeruginosa* (Biedenbach et al., 2015; Livermore et al., 2017; Sader et al., 2017a; Groft et al., 2021; Jean et al., 2021; Liu et al., 2021; Zhang et al., 2021a). In contrast, cefiderocol, a novel siderophore modified from ceftazidime, exhibited excellent *in vitro* activity against several carbapenem-NS GNB species, including *A. baumannii* complex (Hsueh et al., 2019); however, compared to the best available therapy, higher all-cause mortality rates were observed during the early hospitalization course when cefiderocol was administered for the treatment of patients with nosocomial pneumonia and BSI caused by CR-*Acinetobacter* species (Bassetti et al., 2021). The combination of sulbactam with durlobactam has been shown to effectively inhibit CR-*Acinetobacter* species (Shapiro et al., 2021). In addition to cefiderocol (Sader et al., 2017b) and eravacycline (a novel fluorocycline agent of the tetracycline family) (Jean et al., 2019; Jean et al., 2022), several novel drugs and new combination regimens, including TP-6076 (a fully synthetic fluorocycline antibiotic under development), WCK 4234 (a structural DBO β-lactamase inhibitor) with meropenem, LN-1-255 (a β-lactamase inhibitor modified from the penicillanic acid sulfone), taniborbactam, SPR741 (a cationic peptide derived from polymyxin B as an antibiotic adjuvant), and phage therapy (mostly applied in animal models for the treatment of infections caused by *A. baumannii* complex and *P. aeruginosa*) (Pires et al., 2015; Yin et al., 2017), *etc*., have been investigated to evaluate their feasibility for clinical use and potential efficacy against troublesome CR/XDR-*A. baumannii* isolates (Isler et al., 2018).

**Table 4** compares the spectra of novel antibiotics against carbapenemase-producing GNB. Additionally, **Table 5** presents the spectra of important carbapenemase inhibitors against various plasmid-mediated carbapenemases in GNB.

**Table 4:** Comparison of spectra among novel antibiotics against carbapenem-resistant Gram-negative bacteria (Enterobacterales species, and *Pseudomonas aeruginosa*).

Antibiotics (doses)*, Ambler β-lactamase classes, & bacterial species	Enterobacterales species				*P. aeruginosa*	References
	Class A	Class B	Class C	Class D		
Ceftazidime-avibactam (4:1) (2.5 g every 8 h)	++++	–	++ to	++++	++ to	Kuo et al., 2021;
			+++^c^		+++	Sader et al., 2017b
Cefepime-enmetazobactam (AAI101; 2:1) (1.5 g every 8 h)	++^a^	–	+++	+++	±	Papp-Wallace et al., 2019; Morrissey et al., 2019;
						Jean et al., 2022
Cefepime-zidebactam (formerly WCK 5222; 2:1) (3 g every 8 h)	++++	++++	++++	++++	+++ to	Karlowsky et al., 2020;
					++++	Kuo et al., 2021;
						Sader et al., 2017a;
						Jean et al., 2022
Imipenem/cilastatin-relebactam (4:1) (1.25 g every 6 h)	++++	–	++++	–	+++ to	Kuo et al., 2021;
					++++ (porin loss, up-regulated efflux)	Tselepis et al., 2020
Meropenem-vaborbactam (1:1) (4g every 8 h)	++++	–	++++	–	+++	Kuo et al., 2021;
						Novelli et al., 2020
Ceftolozane-tazobactam (2:1) (1.5-3.0 g every 8 h)	+^b^	–	+	–	++++ (efflux)	Jean et al., 2021;
						Hong et al., 2013
Cefiderocol (2 g every 6 h)	++++	++++	++++	++++	++++ (efflux)	Hsueh et al., 2019;
						Sader et al., 2017a;
						Yamano, 2019
Aztreonam-avibactam (3:1) (2 g every 6 h)	++++	++++	+++	++++	MIC_90_, 32 mg/L	Wang Y. et al., 2020 Karlowsky et al., 2017
					(overall, MBL+)	
Omadacycline (100 mg once daily after 200 mg loading dose intravenously, or: 300 mg once daily after 450 mg loading dose orally)	±	–	–	–	–	Jean et al., 2019;
						Jean et al., 2022
Eravacycline (1 mg/kg every 12 h)	++++	++++	++++	++++	–	Jean et al., 2019;
						Jean et al., 2022

+ to ++++ denote the in vitro activity degrees of various drugs against isolates of P. aeruginosa and diverse Types of Ambler β-lactamases in Enterobacterales species, contrasting with – denoting no activity, and ± denoting partial activity against the isolates of interest. MBL, metallo-β-lactamase.

*Doses are recommended for patients with normal creatinine clearance rates.

a Primarily active against producers of extended-spectrum β-lactamase (ESBL) in Enterobacterales species and Klebsiella pneumoniae carbapenemase-producing Escherichia coli.

b Primarily active against ESBL-producing Escherichia coli.

c Less active against naturally inducible chromosomally mediated AmpC-producing carbapenem-resistant Enterobacterales spp. (especially, Enterobacter cloacae complex) than other Enterobacterales species.

**Table 5:** Spectra of important carbapenemase inhibitors against various carbapenemases on Gram-negative bacteria.

Carbapenemase inhibitors	Carbapenemases						References
	Class A	Class B			Class D		
	KPC	NDM	VIM	IPM	OXA-23/24/40	OXA-48/181-like	
Diazabicyclooctane derived							
Avibactam	+	–	–	–	–	+	Kuo et al., 2021;
							Hsueh et al., 2019;
							Sader et al., 2017b
Zidebactam	+	+	+	+	–	+	Kuo et al., 2021;
							Sader et al., 2017a; Livermore et al., 2017;
							Jean et al., 2022
Relebactam	+	–	–	–	–	–	Kuo et al., 2021;
							Tselepis et al., 2020;
							Zhang et al., 2021a
Nacubactam	+	+^a^	+^a^	–	–	+	Mushtaq et al., 2019; Hagihara et al., 2021;
							Barnes et al., 2019;
							Asempa et al., 2020
Durlobactam	+	–	–	–	+	+	Shapiro et al., 2021
Boronic acid derived							
Vaborbactam	+	–	–	–	–	–	Novelli et al., 2020
Taniborbactam	+	+	+	–	–	+	Wang X. et al., 2020
Penicillanic acid sulfone							
Enmetazobactam	± ^b^	–	–	–	–	+	Papp-Wallace et al., 2019; Morrissey et al., 2019; Tselepis et al., 2020;
							Jean et al., 2022

KPC, Klebsiella pneumoniae carbapenemase; NDM, New Delhi metallo-β-lactamase; VIM, Verona integron-encoded metallo-β-lactamase; OXA, oxacillinase; +, active; ±, partially active; -, inactive.

a Active in vitro against isolates limited to Escherichia coli and Enterobacter species for nacubactam alone.

b Active against isolates of solely KPC-producing E. coli.

## Summary

In the present MDR/XDR-GNB era, we have encountered an antibiotic pipeline scenario. Although a few novel antibiotics have been effective *in vitro* against several CR-GNB, their clinical efficacy requires further validation. The judicious prescription of these valuable antibiotics, strict implementation of antibiotic stewardship policy, adequate disinfection of equipment and environment of hospital and LTCF settings, in combination with in-time screening to initiate necessary cohort isolation, are a few measures that must be vigorously undertaken to lessen the rapidly worsening trends of resistance in clinically important GNBs.

## Author Contributions

S-SJ and P-RH collected and analyzed the data. S-SJ and DH participated in writing the manuscript. S-SJ, DH, and P-RH read and approved the final version of the manuscript. All authors contributed to the manuscript and approved the submitted version.

## Conflict of Interest

The authors declare that the research was conducted in the absence of any commercial or financial relationships that could be construed as a potential conflict of interest.

## Publisher’s Note

All claims expressed in this article are solely those of the authors and do not necessarily represent those of their affiliated organizations, or those of the publisher, the editors and the reviewers. Any product that may be evaluated in this article, or claim that may be made by its manufacturer, is not guaranteed or endorsed by the publisher.
